# Highly Selective Aptamer‐Molecularly Imprinted Polymer Hybrids for Recognition of SARS‐CoV‐2 Spike Protein Variants

**DOI:** 10.1002/gch2.202200215

**Published:** 2023-03-20

**Authors:** Mark V. Sullivan, Francia Allabush, Harriet Flynn, Banushan Balansethupathy, Joseph A. Reed, Edward T. Barnes, Callum Robson, Phoebe O'Hara, Laura J. Milburn, David Bunka, Arron Tolley, Paula M. Mendes, James H. R. Tucker, Nicholas W. Turner

**Affiliations:** ^1^ Leicester School of Pharmacy De Montfort University The Gateway Leicester LE1 9BH UK; ^2^ School of Chemical Engineering University of Birmingham Edgbaston Birmingham B15 2TT UK; ^3^ School of Chemistry University of Birmingham Edgbaston Birmingham B15 2TT UK; ^4^ The Aptamer Group Windmill House Innovation Way Heslington York, YO10 5BR UK

**Keywords:** aptamers, molecular imprinting, nanoparticles, SARS‐CoV‐2, viral detection, viral variants

## Abstract

Virus recognition has been driven to the forefront of molecular recognition research due to the COVID‐19 pandemic. Development of highly sensitive recognition elements, both natural and synthetic is critical to facing such a global issue. However, as viruses mutate, it is possible for their recognition to wane through changes in the target substrate, which can lead to detection avoidance and increased false negatives. Likewise, the ability to detect specific variants is of great interest for clinical analysis of all viruses. Here, a hybrid aptamer‐molecularly imprinted polymer (aptaMIP), that maintains selective recognition for the spike protein template across various mutations, while improving performance over individual aptamer or MIP components (which themselves demonstrate excellent performance). The aptaMIP exhibits an equilibrium dissociation constant of 1.61 nM toward its template which matches or exceeds published examples of imprinting of the spike protein. The work here demonstrates that “fixing” the aptamer within a polymeric scaffold increases its capability to selectivity recognize its original target and points toward a methodology that will allow variant selective molecular recognition with exceptional affinity.

## Graphical abstract

A new aptamer specific for wild‐type SARS‐CoV‐2 spike protein is developed and incorporated into a molecularly‐imprinted polymer (MIP) nanoparticle. This hybrid outperforms both individual components (aptamer and MIP) with an equilibrium binding constant below that of the SARS‐CoV‐2 spike—ACE2 receptor interaction; and with clearly superior variant selectivity suggesting a new way to rapidly develop variant selective recognition nanomaterials.

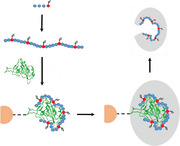

## 1 Introduction

The COVID‐19 pandemic, caused by the novel severe acute respiratory syndrome coronavirus SARS‐CoV‐2, created a global health care emergency with ongoing infections and public health measures. First discovered in December 2019 in Wuhan, China,^[^ ^1^ ^]^ the World Health Organization (WHO) officially declared it a pandemic on the 12th of March 2020; with the infection spreading rapidly across the globe.^[^ ^2^ ^]^ Between then and time of publication multiple variants have emerged.

Named for their surface crown‐like protein spikes, Coronaviruses are enveloped, positive‐stranded RNA (+RNA) viruses with a single‐stranded genome that is among the longest known from RNA viruses.^[^ ^3^ ^]^ Encoded within the genome of SARS‐CoV‐2 are sequences for four structural proteins: envelope (E), membrane (M), nucleocapsid (N), and spike (S). The S protein is a type I fusion protein that forms trimers on the viral surface. These S proteins interact with the angiotensin‐converting enzyme 2 (ACE‐2) receptor on the target cell surface, leading to cellular uptake. For the wild type SARS‐CoV‐2, the equilibrium dissociation constant (K_D_) between the S protein and ACE‐2 receptor is reportedly between 4.7 and 14.7 nM.^[^ ^4^, ^5^, ^6^ ^]^

As with all viruses, genetic mutation is a natural reproductive process and helps to maintain infectivity. Since the original outbreak, there have been multiple reported mutations within the SARS‐CoV‐2 S‐protein, with several variants becoming prominent as these mutations offered evolutionary advantage.^[^ ^7^, ^8^, ^9^ ^]^ As new mutant generation can give rise to different characteristics that may affect diagnostic and vaccine performance, ongoing monitoring of viral variants is critical. The Centre for Disease Control classify these viral variants as variants being monitored, variants of interest, and variants of concern (VOC).^[^ ^10^ ^]^ These classifications are fluid as new variants emerge and supersede existing variants—for example classification of the Delta and Omicron variants superseded prior mutants (Alpha, Beta, etc.)

Polymerase Chain Reaction (PCR) remains the gold standard for the detection and classification of SARS‐CoV‐2 infection. However, molecular recognition agents have proven helpful for in situ testing, such as the use of antibodies in lateral flow tests (LFT). For these tests, the analytical selectivity and specificity provided by the internal affinity reagents are critical to the test performance.^[^ ^11^ ^]^ Significant mutation of the target viral protein may lead to changes in target recognition by the affinity reagents and a resulting reduction in the sensitivity or accuracy of the LFTs.^[^ ^12^ ^]^ It is known that as variants mutate away from the original wild‐type, the capabilities of certain types of detection (molecular testing) are reduced, leading to potential false‐negatives.^[^ ^13^ ^]^ Further critical parameters to LFT performance include stability of the affinity ligand to temperature fluctuations (as seen during storage and transport), as these changes may result in degradation of the internal test antibodies, potentially leading to false negative results. Additionally, the fragile nature of antibodies can limit their use in some field‐testing applications, such as water testing and air monitoring. Therefore alternative, more robust binding agents need to be explored. In particular the ability to generate new, non‐biological molecular recognition elements capable of differentiating between viral protein variants could offer several advantages in terms of development speed, storage and logistical improvements.

Aptamers are short, synthetic, single‐stranded DNA or RNA molecules that are proven alternative affinity reagents for targeting small molecules and proteins alike.^[^ ^14^, ^15^ ^]^ These synthetic oligonucleotide molecules form self‐complementary three‐dimensional structures that allow specific binding to their targets through various non‐covalent interactions. Benefits of aptamers compared with antibodies include their smaller size, low‐cost manufacture, rapid development time, relative ease of modification, and the potential to develop aptamers against non‐immunogenic or toxic targets. In addition, these synthetic reagents have excellent batch‐to‐batch consistency and long shelf life; however, without sequence modification, they are susceptible to degradation under some assay conditions (such as the presence of nuclease enzymes and extremes of pH).

Molecularly imprinted polymers (MIPs) offer another option for robust molecular recognition. Creating a MIP involves the self‐assembly of functional monomers around the template molecule to form a complex, which is entrapped within a large polymer matrix. Removal of the template leaves behind a complementary (steric and chemical) binding pocket. MIPs have been previously limited by high levels of heterogeneity and laborious synthetic manufacturing methods,^[^ ^16^ ^]^ but with substantial advances in molecular modelling and increased understanding and control of the polymer chemistry and nanochemistry (e.g., development of nanoMIPs),^[^ ^17^ ^]^ they have overcome many of these issues.^[^ ^18^ ^]^ Though MIPs have traditionally favored small molecule targets, protein imprinting, acknowledged to be more complex, is rapidly expanding with a number of different methods being actively researched.^[^ ^19^ ^]^

Recently, hybrid materials, that is, materials that utilize both aptamer and MIP properties, have been developed.^[^ ^20^ ^]^ Jolly et al. demonstrated a method using an electrochemical sensor to detect PSA using an aptamer as a capture agent within an imprinted film,^[^ ^21^ ^]^ while Rad demonstrated similar abilities with tetracycline.^[^ ^22^ ^]^

**Figure 1:**
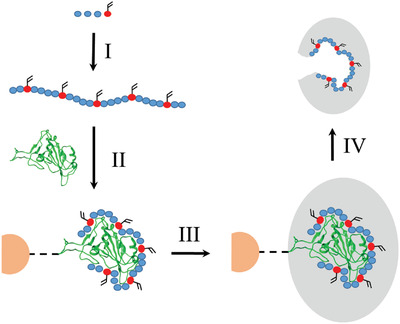
Schematic representation of the solid‐phase synthesis of aptaMIP NPs. Red circles indicate the modified polymerizable base, blue circles indicate normal bases, orange semi‐circle indicate solid support, and the green ribbon represents the spike protein subunit template molecule. I) Synthesis of aptamer sequence to include polymerizable moieties. II) Complexation of aptamer with subunit target moiety attached to an inert solid phase. III) Addition of polymer scaffold components, polymerization, and formation of polymer scaffold. IV) Thermal (60 °C) release of the aptamer incorporated nanoparticle. Notes: 1) The spike protein subunit template is left affixed to support for re‐use. 2) Non‐aptamer bearing control nanoMIPs made using the same solid‐phase method as shown, but without the aptamer present. 3) Where an epitope has been used as a template it replaces the green ribbon. 4) Aptamer sequence is not to scale, but is representative of method.

Our group has taken this work a stage further: through direct modification of the aptamer sequence, by incorporation of polymerizable groups on modified thymine residues, aptamers can be directly and covalently incorporated into the polymer matrix (**Figure** **1**). This has been successfully demonstrated for small molecule,^[^ ^23^, ^24^ ^]^ nucleic acid sequence,^[^ ^25^ ^]^ and protein targets,^[^ ^26^ ^]^ using a nanoparticle solid‐phase synthetic method. The developed apta‐MIP reagents have previously shown improved performance (in terms of both K_D_ and selectivity) over both constituent parts, with an average of a tenfold improvement over standard nanoparticle MIP (without the aptamer present) and up to 100‐fold improvement over the aptamer recognition element alone, when sensing the antibiotic moxifloxacin.^[^ ^23^ ^]^

MIPs to the SARS‐CoV‐2 S protein have been imprinted in several formats.^[^ ^27^ ^]^ Bognár et al. used an epitope process to imprint films onto a surface plasmon resonance (SPR) sensor. A novel spotting method was used to deposit the epitope, followed by an electropolymerized layer to form the imprint. The sensor demonstrated nM K_D_ values for the receptor binding domain (RBD) of the protein and, when using virus‐like particles, estimated fM equilibrium constants.^[^ ^28^ ^]^ Similarly, Syrityski's group demonstrated an electrochemical approach using surface imprinting^[^ ^29^ ^]^ that performed well compared to existing electrochemical techniques. Using this method, selectivity was shown to be possible between different protein targets, but distinguishing between SARS‐CoV‐2 viral variants was not possible. Further testing on clinical samples revealed some variation in performance.

Puoci proposed the use of imprinted nanoparticles,^[^ ^30^ ^]^ which has been considered by several groups and represents the core of this study. The same group used a microemulsion polymerization method to develop nanoparticles that were able to inhibit viral replication.^[^ ^31^ ^]^ The Peeters group alongside MIP Discovery demonstrated a nanoMIP with a K_D_ values of 7–18 nM which offered fg mL^−1^ level detection using a thermal detection measurement system. Here the different variants studied showed similar levels of detection.^[^ ^32^, ^33^ ^]^

In this work, we have explored the ability of different nanomaterials to potentially differentiate between variants of the SARS‐CoV‐2 spike protein. Using the wild type SARS‐CoV‐2 S protein as a target for affinity ligand generation, we compared the reagent's recognition via affinity and selectivity against known mutations (from early stages of the pandemic) and against other coronavirus spike proteins. We have developed four new entities: a new aptamer; a nanoMIP imprinted against the whole spike protein; a nanoMIP against a selected epitope from within the RBD; and an aptaMIP combining our new aptamer and nanoMIP methodologies. Finally, the performance of these reagents in terms of target recognition is compared, and we discuss the capabilities of these technologies to distinguish between SARS‐CoV‐2 S protein variants.

## 2 Results and Discussion

Using an automated selection method, a truncated DNA aptamer (commercially known as an Optimer binder) was generated against the S1 domain of the wild‐type (WT) SARS‐CoV‐2 spike protein through 8 successive rounds of selection and preferential amplification. This was carried out by the team at Aptamer Group. The final optimized sequence (protected for commercial reasons) was 33 bases long (5′‐**T***T********T***T*******T***T*‐3′) with the 6 thymines shown in bold chosen as modification sites for aptaMIP generation. The thymines are distributed throughout the sequence, giving multiple binding points for covalent incorporation into a polymer backbone.

**Figure 2:**
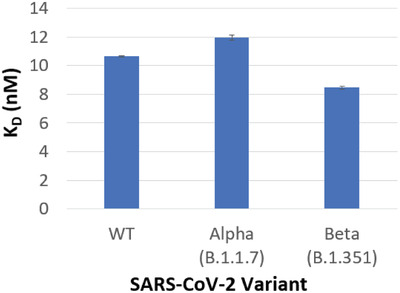
Equilibrium dissociation constants (K_D_) of identified SARS‐CoV‐2 aptamer to SARS‐CoV‐2 WT and variants of concern, determined via BLI. *N* = 5.

During the development process, the selectivity of the identified SARS‐CoV‐2 aptamer was assessed using Bio‐Layer Inferometry (BLI) by comparing binding affinities with recombinant S1 protein domains of the homologous coronaviruses SARS‐CoV, MERS‐CoV, and SARS‐CoV‐2 (data not shown). Due to the continuous evolution of the SARS‐CoV‐2 virus, interaction analysis was also performed to determine its binding affinities to the S1 domain of SARS‐CoV‐2 VOC. The truncated aptamer (Optimer) exhibits nanomolar binding affinities toward spike (S1) proteins derived from the SARS‐CoV‐2 WT virus (10.64 +/− 0.04 nM), as well as, the Alpha (11.96 +/− 0.18 nM), and Beta (8.46 +/− 0.12 nM) variants (**Figure** **2**). The curves associated with this data are shown in Figure S1, Supporting Information. Interestingly, the three values are very similar, suggesting a similar folded conformation interaction with each target, despite the mutations, with some potential flexibility in aptamer conformation. Cross‐reactivity of this SARS‐CoV‐2 S1 aptamer was also tested with SARS‐CoV S1 and MERS‐CoV S1. In each case, the aptamer was shown to be highly specific to SARS‐CoV‐S2 (Figure S2, Supporting Information). This matches the selectivity against the SARS and MERS subunits observed in development process.

With an effective selective aptamer displaying excellent affinity established, we looked to explore its incorporation into a polymeric scaffold. The aim of this was to investigate whether the “aptaMIP” process increased the affinity and stability of the aptamer, these parameters being important for potential environmental and clinical sensor applications. This innovative strategy, adapted from our previous work,^[^ ^23^, ^26^ ^]^ was utilized for the synthesis of water‐soluble hybrid aptamer‐MIP nanoparticles for the SARS‐COV‐2 S1 subunit (known henceforth as the aptaMIP). The 33‐mer active sequence as described above (redacted for commercial sensitivity) was produced, with several carboxyvinyl polymerizable functional groups integrated into the thymine residues. This method, enabling covalent attachment of a nucleic acid sequence into a polymeric scaffold, has been shown previously to be capable of “fixing” an aptamer into a favored conformation, thus increasing its affinity.^[^ ^23^, ^24^, ^26^ ^]^ Based on this prior work, we decided to modify all 6 thymines on the aptamer with anchoring points to provide an optimal binding performance over single‐point linkers at the 5′ or 3′ ends.^[^ ^24^, ^26^ ^]^

Two control MIP nanoparticles (nanoMIPs) to compare against the aptaMIP were also made, using a method adapted from Safaryan et al.^[^ ^34^ ^]^ This was achieved by following the same process as described in Figure 1, but the aptamer sequence was not added to the standard polymerization mixture.

The first control (protein nanoMIP) was targeted at same WT spike protein subunit as used for the aptaMIP synthesis. The second (epitope nanoMIP) was targeted at an epitope PCNGVEGFNC (positions 479–488 of the spike protein). This epitope was selected as it is part of the receptor binding motif within the RBD of the spike protein. The accessible nature of this sequence, along with the finding that it has been shown to be important in interactions between the ACE‐2 receptor and the spike protein, made it a suitable candidate for an imprinting target.^[^ ^4^ ^]^ A second short peptide sequence GGC was attached to this epitope sequence to enable attachment either to a solid phase through 1‐ethyl‐3‐(3‐dimethylaminopropyl)carbodiimide (EDC)/NHS chemistry (via a terminal COOH) or, if required, directly onto a gold surface (via the thiol residue on the cysteine). Two glycine residues were used as minimal small non‐functionalized R‐group spacers to ensure the epitope was more accessible and less sterically hindered by solid‐phase attachment.

From the synthesis above, the concentration of the aptaMIP, protein nanoMIP and epitope nanoMIP nanoparticles were calculated to be 270 ± 12.4 µg mL^−1^, 253.3 ± 7.1 µg mL^−1^ and 330 ± 22.6 µg mL^−1^, respectively. These concentrations were calculated by incubating 3 mL of the resultant sample at 60 °C until dry, measuring the particle mass and calculating the concentration (per mL). These concentrations are within the yield levels expected based on earlier reports from our group and others.^[^ ^23^, ^26^ ^]^

Dynamic light scattering (DLS) was used to size the materials. The diameters observed were for the aptaMIP (117.2 ± 9.2 nm), protein nanoMIP (102.4 ± 8.4 nm), and epitope nanoMIP (72.8 ± 7.4 nm) in water at 25 °C. The DLS curves (Figure S3, Supporting Information) show excellent Gaussian distributions, confirming that the production protocol creates homogenous particles with acceptable size ranges. The slight difference in size between the aptaMIP and the protein nanoMIP is consistent with previous work^[^ ^23^, ^26^ ^]^ and can be ascribed to the aptamer interacting with the protein template as intended before the subsequent polymerization process (Figure 1). This would be expected to offer a superior (larger) nucleation site compared to the random orientation of monomers in the polymerization of the nanoMIP. This phenomenon is currently under further investigation as it could be important in the optimization of the design of this new class of nanomaterial. The smaller diameter of the epitope nanoMIP can be readily explained by the smaller size of the templating molecule.

### 2.1 Binding Performance of the Aptamer‐Molecularly Imprinted Polymers and nano‐Molecularly Imprinted Polymers

200 µg of each of the synthesized imprinted nanoparticles (aptaMIPs and both nanoMIPs) were individually dissolved in Phosphate Buffered Saline running buffer (PBST), in the presence of sodium acetate (to activate the amine functional groups within the polymer matrix), and covalently deposited onto a gold Surface Plasmon Resonance) SPR chip, coated with a carboxymethyl dextran hydrogel layer (whose —COOH functional groups were activated using NHS and EDC). Residual unreacted carboxyl groups were quenched by an injection of ethanolamine and washed to remove any unbound nanoparticles. Due to the EDC/NHS coupling chemistry used in the immobilization of the amine functionalized nanoMIPs to the SPR chip surface, a monolayer of the nanoMIPs is expected to be deposited on the surface, as the polymers will bind only to the surface, and not to themselves (see Figure S4, Supporting Information, schematic). Adding the initial deposition of nanoMIPs in excess, in a relatively slow flow, achieves maximal coverage on the chip. Given the nature of this deposition reaction, a single layer of nanoparticles is achieved. These bound nanoparticles were then challenged with a range of analytes at 5 different concentrations.

**Figure 3:**
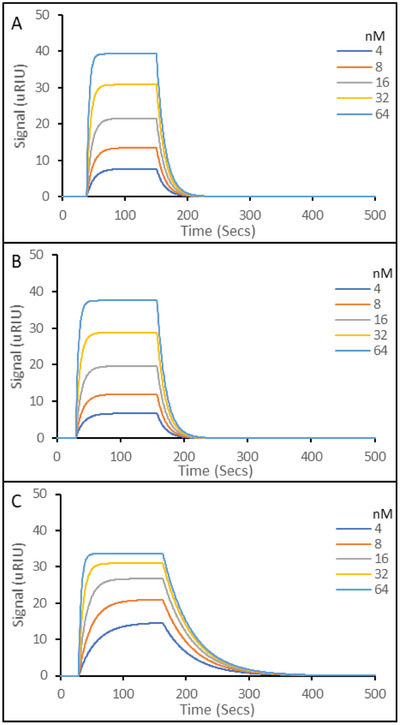
Representative SPR sensorgrams of molecular interactions of various nanoparticles immobilized on carboxymethyl dextran hydrogel coated Au chips, to solutions containing 5 concentrations of SARS‐CoV‐2 Spike protein S1 subunit. A) SARS‐CoV‐2 Spike protein S1 Subunit binding to aptaMIP. B) SARS‐CoV‐2 Spike protein S1 Subunit binding to protein nanoMIP. C) SARS‐CoV‐2 Spike protein S1 Subunit binding to epitope nanoMIP. The SPR running buffer (PBST) was a phosphate buffered saline made at 10 mm, pH 7.4, supplemented with 0.01% (v/v) Tween 20. Tween 20 is included to reduce non‐specific binding during rebinding studies. Regeneration buffer was 10 mm Glycine‐HCl at pH 2. All rebinding at 25 °C. All experiments in triplicate.

**Figure 4:**
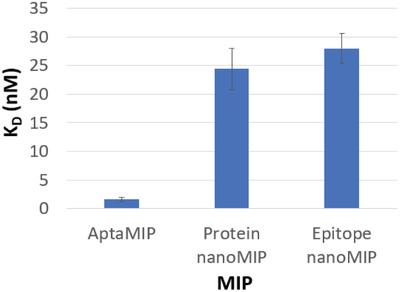
Equilibrium dissociation constants (K_D_) of analyzed polymers toward the SARS‐CoV‐2 WT S1 protein (Example data shown in Figure 3). *N* = 3.

**Figure** **3** shows representative SPR sensorgrams for the three different imprinted polymers aptaMIP (3A), protein nanoMIP (3B), and epitope nanoMIP (3C) with the SARS‐CoV‐2 Spike protein S1 subunit. The SPR sensorgrams show a sharp and complete dissociation of the target analyte, which is consistent with our previous work.^[^ ^23^, ^26^, ^35^, ^36^ ^]^ This shape of curve is often observed with antibody‐analye interactions and highlights the “on/off” nature of strong association/dissociation profile in highly specific host/guest models. The comparative calculated equilibrium constants for the nanoparticles (from triplicate experiments) are shown in **Figure** **4**.

The protein nanoMIP and epitope nanoMIP polymers produced in this study have calculated K_D_ values of 24.4 nM and 28.0 nM, respectively for their interactions with the original spike protein (in PBST). This is consistent with prior work,^[^ ^32^, ^33^ ^]^ and is comparable to that of monoclonal antibodies.^[^ ^37^ ^]^ It is also in the same order of magnitude (10^−8^ M) as the aptamer generated in this study.

The calculated K_D_ value for spike protein rebinding onto the epitope nanoMIP (28.0 nM) is comparable to that of the calculated K_D_ value for spike protein rebinding onto the protein nanoMIP (24.4 nM). This validates the selection of the epitope, and highlights that imprinting the epitope is an attractive alternative to imprinting the whole protein. This strategy reduces cost as protein production and purification is high, their stability is low, and the imprinting process for proteins could potentially be long and complex. Using smaller epitopes allows the focus of the nanoMIP synthesis to be on the primary structure of the peptide instead of the more complex tertiary and quaternary structures of a target protein. This is similar to the use of representative peptide fragments often used to generate epitope selective antibodies.^[^ ^38^ ^]^

The interaction between the original spike protein and the aptaMIP produced a much lower K_D_ value of 1.61 nM (Figure 4). This is an increase in binding affinity compared with protein nanoMIP and epitope nanoMIP (24.4 nM and 28.0 nM, respectively). The ratio of K_D_ values clearly justifies the inclusion of a pre‐target macro‐monomer to improve performance, with a K_D Protein nanoMIP_/K_D aptaMIP_ value of 15.2 and K_D epitope nanoMIP_/K_D aptaMIP_ value of 17.4. This increase in binding strength is consistent with, and an improvement, on our previous work where higher affinities were observed when incorporating aptamers into polymer scaffolds to generate a trypsin aptaMIP (K_D nanoMIP_/K_D aptaMIP_ of 1.80) and a moxifloxacin aptaMIP (K_D Protein nanoMIP_/K_D aptaMIP_ of 13.3), respectively.^[^ ^23^, ^26^ ^]^ This shows that fixing the aptamer into the scaffold of the polymer matrix improves binding, when compared to a nanoMIP, made in the same way but in the absence of the polymerizable DNA aptamer. The K_D aptamer_/K_D aptaMIP_ value of 6.60 when targeting the wild type spike protein shows that the aptaMIP process also improves binding performance against the aptamer only. This is hypothesized to be due to the aptamer being fixed in an optimal binding conformation within the polymer matrix, hence reducing entropic effects (flexing and reorientation of the aptamer).

**Figure 5:**
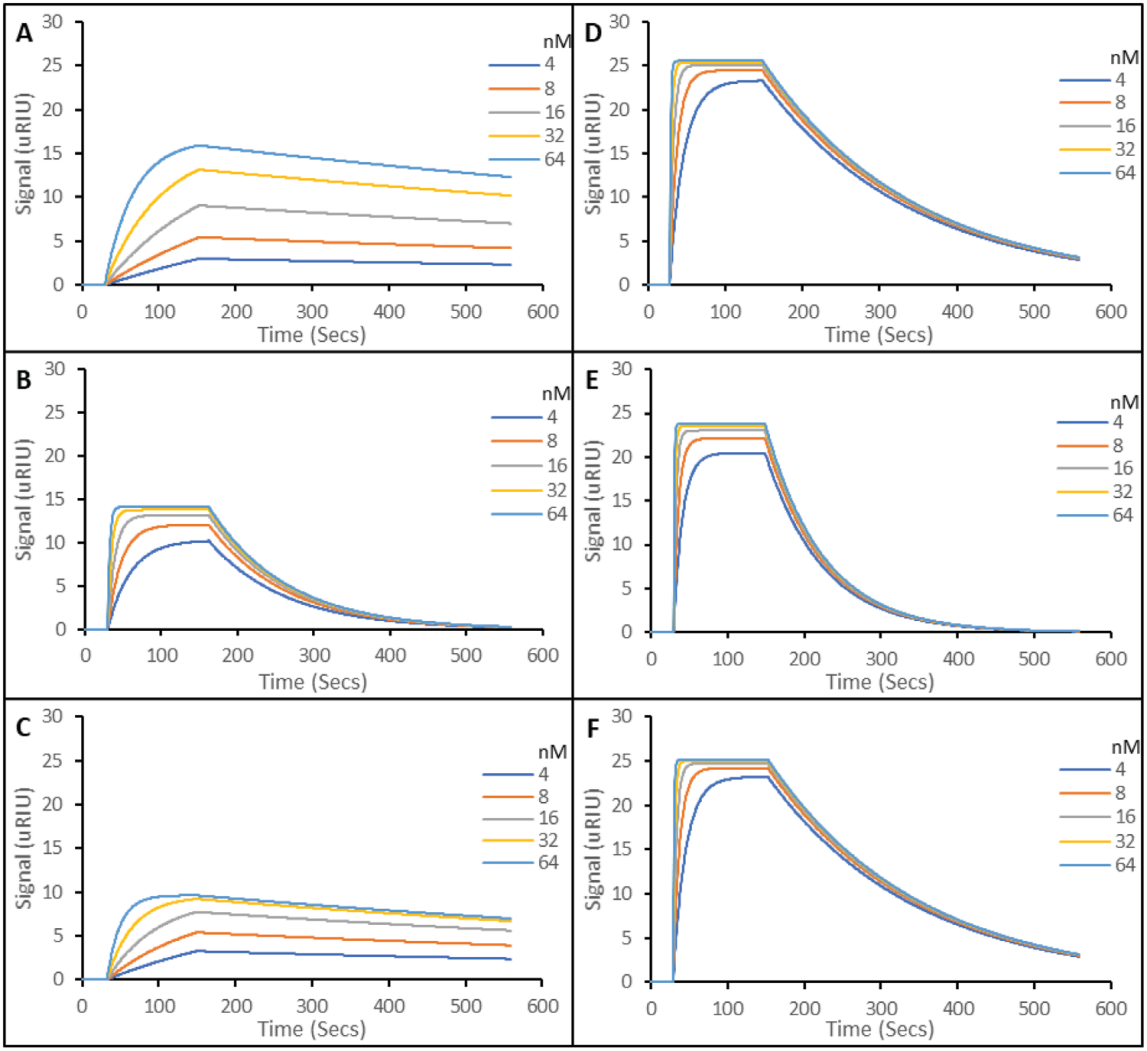
Representative SPR sensorgrams of molecular interactions of various nanoparticles immobilized on carboxymethyl dextran hydrogel coated Au chips, to solutions containing five concentrations of SARS‐CoV‐2 Spike protein S1 subunit. A) MERS‐CoV binding to the aptaMIP. B) MERS‐CoV binding to the protein nanoMIP. C) MERS‐CoV binding to the epitope nanoMIP. D) SARS‐CoV binding to the aptaMIP. E) SARS‐CoV binding to the protein nanoMIP. F) SARS‐CoV binding to epitope nanoMIP. The SPR running buffer (PBST) was a phosphate buffered saline made at 10 mm, pH 7.4, supplemented with 0.01% (v/v) Tween 20. Tween 20 is included to reduce non‐specific binding during rebinding studies. Regeneration buffer was 10 mm Glycine‐HCl at pH 2. All rebinding at 25 °C. All experiments in triplicate.

With sensitivity established, the next task was to evaluate specificity. This was done by examining cross‐reactivity and non‐specific binding, using the non‐target spike proteins SARS‐CoV^[^ ^39^ ^]^ and MERS‐CoV^[^ ^40^ ^]^ (**Figure** **5**). Although these targets share some characteristics with SARS‐CoV‐2,^[^ ^4^, ^41^ ^]^ they are sufficiently structurally dissimilar for good selectivity to be demonstrated here in all three cases, with K_D_ values in the 10^−7^ to 10^−6^ M range (summarized in **Table** **1**).

**Table 1:** Summary of all calculated equilibrium dissociation constant (K_D_) of imprinted materials; All of these experiments were repeated in triplicate and the SPR curves were fitted to a 1:1 interaction model

	K_D_ [nM]		
Variant	AptaMIP	Protein nanoMIP	Epitope nanoMIP
Wild type spike protein	1.61 (±0.4)	24.4 (±3.6)	28.0 (±2.6)
Alpha (B.1.1.7)	24.9 (±2.1)	59.0 (±1.5)	65.2 (±5.9)
Beta (B.1.351)	8.57 (±0.7)	24.2 (±6.4)	30.4 (±2.8)
SARS‐CoV	359 (±42)	411 (±29)	1050 (±110)
MERS‐CoV	860 (±71)	700 (±31)	1540 (±630)

In terms of spike protein sequence, SARS‐CoV has a closer structural similarity to the WT SARS‐CoV‐2 protein than MERS‐CoV. This is consistent with the binding data, where MERS‐CoV binds less strongly than SARS‐CoV, though some non‐specific binding is observed as is common for MIPs. This arise through interactions with the polymer matrix outside of the binding pocket. However, this data also highlights a further benefit of applying an aptameric “macromonomer” into the binding pocket, with a K_D SARS‐CoV_/K_D SARS‐CoV‐2_ ratio of 223 for aptaMIP vs 16.8 for protein nanoMIP, demonstrating a clear improvement in selectivity. Interestingly, epitope nanoMIP gives a K_D SARS‐CoV_/K_D SARS‐CoV‐2_ ratio of 37.5, which is better than the imprint from the entire subunit. Given the difference in target sequence in the same epitope loop: PCTP‐PALNC versus PCNGVEGFNC for SARS‐CoV and SARS‐CoV‐2 respectively (highlighted bold residues are altered), this is to be expected. This highlights the benefits of the epitope process, but significantly demonstrates the benefit of the aptameric monomer in improving the imprinting selectivity.

**Figure 6:**
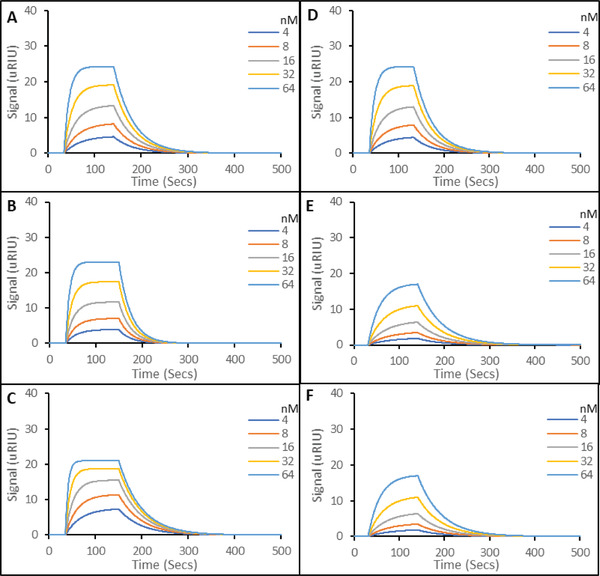
Representative SPR sensorgrams of molecular interactions of various nanoparticles immobilized on carboxymethyl dextran hydrogel coated Au chips, to solutions containing five concentrations of SARS‐CoV‐2 Spike protein S1 subunit. A) Alpha (B.1.1.7) binding to the aptaMIP. B) Alpha (B.1.1.7) binding to the protein nanoMIP. C) Alpha (B.1.1.7) binding to epitope nanoMIP. D) Beta (B.1.351) binding to the aptaMIP. E) Beta (B.1.351) subunit binding to the protein nanoMIP. F) Beta (B.1.351) binding to epitope nanoMIP. The SPR running buffer (PBST) was a phosphate buffered saline made at 10 mm, pH 7.4, supplemented with 0.01% (v/v) Tween 20. Tween 20 is included to reduce non‐specific binding during rebinding studies. Regeneration buffer was 10 mm Glycine‐HCl at pH 2. All rebinding at 25 °C. All experiments in triplicate.

**Figure 7:**
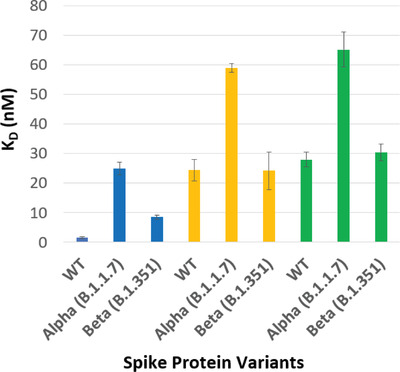
Equilibrium dissociation constants (K_D_) of analyzed polymers toward the studied variants. Blue = aptaMIP; Orange = protein nanoMIP. Green = epitope nanoMIP. *N* = 3.

Molecular imprinting is a technique that relies on both steric and chemical complementary principles, therefore it was interesting to consider whether this selectivity could be used to discriminate between different variants of the same spike protein—a variation in a small number of residues might be expected to offer subtle differences in structure and electrostatics. To study the ability of the nanoparticles to discriminate between different SARS‐CoV‐2 variants, Alpha (B.1.1.7)^[^ ^42^ ^]^ and Beta (B.1.351),^[^ ^43^ ^]^ were captured by the aptaMIP, protein nanoMIP, and epitope nanoMIP (**Figure** **6**). A comparative chart is shown in **Figure** **7**.

The data presented in Figure 7 and summarized in Table 1 shows that all imprinted materials are able to bind different variants of the spike protein. In all cases, the aptaMIP shows the greatest affinity for each of the variants, followed by the whole protein imprint, and then the epitope. It is accepted that the type of molecular recognition present with MIPs is an induced fit so some cross‐reactivity of such similar large structures is expected. Note the difference in the shape of the curves between those of the wild‐type (Figure 3), which exhibit the best selectivity (sharpest association/dissociation), and the non‐template targets in Figure 6 where both association and dissociation are less pronounced. In terms of specificity, the benefits of using the aptamer as a macromonomer is once again clearly demonstrated. The selectivity ratio of K_D Alpha_/K_D WT_ is ≈15 for aptaMIP, while it is only ≈2 for both the protein nanoMIP and epitope nanoMIP. The same pattern is observed for the Beta variant, though to a lesser, but still significant degree where only the aptaMIP is able to distinguish between variants (aptaMIP = ≈5 vs ≈1 for both nanoMIPs). This highlights the significant potential here to develop variant‐specific imprints capable of recognizing individual variants through the aptaMIP process.

Key to the idea that it is possible to create variant specific imprints is the differences between the aptamer data on its own (Figure 2) and when it is embedded in the polymer scaffold (Table 1). When on its own, the aptamer shows the ability to recognize the variants with approximately equal affinities, suggesting that the aptamer has sufficient flexibility to alter its binding conformation as appropriate; however, when entrapped into a set binding conformation through the imprinting process, significant differences in affinities begin to emerge. We can hypothesize that this is due to: a) The aptamer no longer having the ability to alter its conformation as it is fixed throughout; and/or b) the additional steric recognition effect (shape specific binding pocket) afforded by the proximate MIP polymer scaffold—it is likely that a combination of both reduces the relative the affinities for the two variants. The latter shape is observed in the two nanoMIP systems (protein and epitope), which perform to a reasonable extent, but only when the aptamer is added does the system really demonstrate it's potential.

Overall the data suggests that increased variant specificity is possible, suggesting that through further tailoring (e.g., using variant‐specific aptamers, molecular modelling, and/or alternative monomer composition) materials with selectivity for particular variants can be developed with exceptional affinity, with all the benefits of the stability granted by the MIP process.

## 3 Conclusion

In this study we have successfully demonstrated the synthesis of a new SARS‐CoV‐2 aptamer, two nanoscale MIPs that target the SARS‐CoV‐2 spike protein subunit; and a hybrid aptamer‐MIP material (an aptaMIP) that incorporates the biorecognition features of both. The aptaMIP displays superior performance compared to its separate components. The production of these hybrid materials is relatively straightforward, utilizing accepted SELEX technology, and an adapted solid‐phase MIP synthesis methodology. The gentle polymerization conditions employed allow for both the aptamer monomers and protein template to retain their activities, thus removing the possibility of denaturation during the polymerization process.

These hybrid spike protein binders could lead the way for potential sensor applications for novel virus detection. The improvements in binding affinity demonstrated by these materials are comparable to those of monoclonal antibodies (which offer *K*_D_ values in the pM/nM range) making them potential substitutes for antibodies in lateral flow systems. We are currently exploring the use of these materials as the recognition element in an SPR‐based sensor platform with a range of relevant samples, under different environmental and physiological conditions (temperature and pH).

Excitingly, we have shown that these aptaMIP materials offer excellent and consistent recognition toward the Covid‐19 spike protein, with exceptional specificity toward the WT imprinted variant retained, which is essential when new variants emerge. The potential of mutations and variants escaping regular detection (via antibodies) is of great concern and this work offers a potential way to develop a rapid response technology that can be achieved in a matter of weeks. The simplicity and ease of use for this robust synthetic methodology, supported by our previous work, means that new aptaMIP materials could be quickly produced for any new variants or new viruses.

Furthermore, with K_D_ values comparable to those obtained using monoclonal antibodies, the opportunity to use these high performing aptaMIPs within virus neutralizing therapies is now a possibility. Taylor et al. showed that monoclonal antibodies have promising potential for Covid‐19 treatment,^[^ ^44^ ^]^ combined with the work of Graham et al., who showed that a hydrogel‐based MIP was capable of specifically neutralizing infectivity in vitro.^[^ ^45^ ^]^ This indicates the potential of our aptaMIPs to specifically neutralize virus activity and to be clinically relevant. Puoci and co‐workers have previously shown the potential of nanoMIPs to inhibit ACE‐2—spike protein interactions.^[^ ^31^ ^]^ Given that our data shows affinities between the aptaMIP and spike protein that are greater than (lower K_D_ that of the ACE‐2/spike protein interaction, with added variant selectivity there is significant potential for therapeutic applications to be readily developed, especially given the relatively straightforward and rapid nature of the aptaMIP design and synthesis stage.

This work also demonstrates the strength of the imprinting process in general. While antibodies still occupy the mainstream, MIPs have proven themselves to be commercially successful with several companies exhibiting excellent MIP‐based products and application of MIPs are growing. This means that routes to market for this technology exist and are being explored.

## 4 Experimental Section

### Materials and Equipment

EDC, 3‐aminopropyltrimethyloxy‐silane (APTMS), acrylic acid, ammonium persulphate (APS), dipotassium phosphate, disodium phosphate, ethanolamine, ethylenediaminetetraacetic acid, glutaraldehyde (GA), glycine, *N,N*'‐methylenebisacrylamide (BIS), *N*‐hydroxysuccinimide (NHS), *N*‐isopropylacrylamide (NIPAm), *N*‐*tert*‐butylacrylamide (TBAm), tetramethylethyldiamide (TEMED), and trypsin were all purchased and used without purification from Sigma‐Aldrich, Poole, Dorset, UK. Acetone, acetonitrile (dry), methanol, potassium chloride, and sodium hydroxide were all purchased and used without purification from Fisher Scientific UK Ltd, Loughborough, UK. All chemicals and solvents were analytical or high‐performance liquid chromatography (HPLC) grade and were used without further purification. Double‐distilled water was used for the analysis.

Glass beads (75 µm diameter) were purchased from Microbeads AG, (Brugg, Switzerland) and used as supplied. Carboxymethyl Dextran Hydrogel Surface Sensor chips were purchased from Reichert Technologies Life Sciences, Buffalo, New York, USA.

The SPR running buffer (PBST) was a phosphate buffered saline made at 10 mm, pH 7.4, supplemented with 0.01% (v/v) Tween 20. Tween 20 is included to reduce non‐specific binding during rebinding studies. Regeneration buffer was 10 mm Glycine‐HCl at pH 2.

Recombinant SARS‐CoV‐2 Spike Protein, S1 Subunit and recombinant SARS‐CoV‐2 Spike Protein, S1 subunit and host cell RBD were purchased from Cambridge Bioscience (Cambridge, UK). Other spike proteins targets were purchased from Sino Biological, Eschborn, Germany. The epitope sequence was purchased from Pepceuticals (Leicester, UK).

### Aptamer Identification

Aptamers were generated by in vitro selection, from a diverse starting library of 10^14^ different sequences. Aptamer selection was performed by Aptamer Group (York, UK) according to proprietary automated selection methods. These aptamers are commercially known as Optimer binders. Briefly, DNA aptamers were selected against the S1 domain of the SARS‐Cov‐2 Spike protein (Sino Biological, Eschborn, Germany) through 8 successive rounds of selection and preferential amplification. Following identification of the best performing individual aptamer sequences, the minimal functional fragment of the aptamers (the Optimers), were identified and assessed for binding to the SARS‐CoV‐2 S1 domain and the SARS‐CoV‐2 Spike protein trimer (Peak Protein, Macclesfield, UK) by Bio‐Layer Interferometry (BLI) using an Octet Red 384 system (Sartorius, Goettingen, Germany). Cross‐reactivity to the homologous SARS‐CoV, and MERS‐CoV (Sino Biological), was also assessed.

### Assessment of Aptamer Performance Using Bio‐Layer Interferometry

The truncated SARS‐CoV‐2 binding aptamers were synthesized with a biotin modification at the 5′ terminus to allow subsequent conjugation to the streptavidin‐coated biosensor (Octet Red 384, Sartorius, Germany). Briefly, the selected truncated, biotinylated aptamer against wild‐type SARS‐CoV‐2 S1 was immobilized to streptavidin‐coated biosensors, to a loading response between 1.5 and 1.9 nm. The probes were washed and the assay baseline established in an MES‐based selection buffer. Interaction kinetics were then assessed by monitoring the association with each of the respective spike proteins, using an 8‐point, twofold serial dilution series, starting at a concentration 10x above an estimated K_D_ (established during aptamer pool validation); followed by dissociation in the MES‐based assay buffer. The resulting BLI traces were fit to a global 1:1 binding model to determine the dissociation constant (K_D_) of the aptamer for each protein variant. Cross‐reactivity to SARS‐CoV and MERS‐CoV S1 was also determined using immobilized aptamers and monitoring their interaction with 0.2–0.4 µM of each of the respective spike proteins in solution.

### Synthesis of Polymerizable Aptamer Sequence

The Covid‐19 Spike Protein aptamer was synthesized under standard conditions at 1 µmol scale using modified polymerizable T bases (5′‐**T***T********T***T*******T***T*‐3) on an Applied Biosystems 394 oligonucleotide synthesizer. The polymerizable base used in this study was carboxy‐dT, a thymine modified with a carboxyvinyl moiety on the 5′ position. This was used in previous studies and has demonstrated excellent incorporation in the polymer matrix.^[^ ^23^, ^24^, ^26^ ^]^ The synthesized oligomers were deprotected and released from the support by treatment with concentrated aqueous ammonia at 60 °C for 24 h. The solutions were concentrated to dryness, resuspended in water, and purified by semi preparative HPLC on an Agilent 1260 infinity system with a Phenomenex Clarity 5 µm Oligo‐RP LC 250 × 10 mm column. Collected fractions were desalted using NAP‐10 columns (GE Healthcare) and oligo purity was determined by analytical HPLC on an Agilent 1260 infinity system with a Phenomenex Clarity 5 µm Oligo RP LC 250 × 4.6 mm column. Oligonucleotide masses were verified using a Waters Xevo G2‐XS, and concentrations were determined by optical density at 260 nm using a BioSpec‐nano micro‐volume UV‐Vis spectrophotometer (nanodrop, Shimadzu), and the Beer Lambert law, with extinction coefficients obtained from OligoAnalyzer (Integrated DNA Technologies).

### Preparation of Affinity Media

Following previous protocols,^[^ ^18^, ^46^ ^]^ 30 g of glass beads were activated in alkaline conditions, washed and then incubated in 3% (v/v) APTMS in anhydrous toluene overnight at 60 °C. After washing in acetone and methanol and drying, 10 g of the amine‐functionalized beads were then incubated in a 7% (v/v) glutaraldehyde solution (1 mL of solution per gram of beads) for 2 h at room temperature. To 5 g of these GA‐modified beads, either: i) 500 µg of SARS‐COV‐2 S1 Subunit (Spike Protein) in 3 mL PBS solution; or ii) 500 µg of epitope PCNGVEGFNCGGC in 3 mL PBS solution; were sealed under nitrogen, and incubated at room temperature overnight. Derivatized beads were washed thoroughly with doubled‐distilled water and dried under vacuum. After this step, beads were used straight away for the synthesis of nanoMIPs without further storage.

### Solid‐Phase Synthesis of Covid‐19 Spike Protein/Epitope Imprinted Molecularly Imprinted Polymers (NanoMolecularly Imprinted Polymers)

Following previously established protocols, a polymerization mixture consisting of NIPAm (20 mg), BIS (1 mg), and AA (2.2 µL) in 49 mL of double distilled water was produced. In a separate vial 17 mg of TBAm was dissolved in 250 µL of ethanol and this was added to the previous solution and adjusted to 50 mL with water. After degassing, and bubbling with nitrogen, this solution was added to 5 g of selected affinity media in a sealed nitrogen atmosphere bottle under a nitrogen purge. 12.5 µL of TEMED and 15 mg of APS dissolved in 250 µL of double distilled water was added, and left to polymerize for 1 h at room temperature.

After synthesis, the beads were filtered through an 11 µm filter paper, using gravity filtration, and then washed with water aliquots (5 × 20 mL), initially at ambient temperature in order to remove the impurities, unreacted monomers and low‐affinity nanoMIPs, and then with 100 mL (5 × 20 mL) of water at 60 °C (in aliquots) to elute high affinity nanoMIPs. This high affinity nanoMIPs solution was stored at 4 °C.

To make the aptaMIP, the procedure above was followed except 1.74 µmol of the polymerizable modified aptamer was added to the polymerization mixture.

### Physical Characterization of Imprinted Nanoparticles

Effective hydrodynamic diameters (*d*_h_) of the particles were determined by DLS with a NanoBrook Omni spectrometer (Brookhaven, United States) at 25 °C in water, and using Particle Solutions (v2.6) software.

The shape and surface topography of the nanoparticles were determined using a Carl Zeiss SEM EVO High Definition 15 Scanning Electron Microscope (Carl Zeiss, Germany) operating at 15 kV. The samples were mounted on a metal stub with double‐sided adhesive tape and gold‐coated under vacuum in an argon atmosphere prior to observation.

### Surface Plasmon Resonance Analysis of nanoMolecularly Imprinted Polymers/Aptamer‐Molecularly Imprinted Polymer Affinity/Selectivity

A 3 mL aliquot of the nanoparticle solution was dried and weighed, then resuspended as needed allowing for a concentration (in µg mL^−1^) of the solution to be calculated.

The affinity and specificity of the imprinted nanoparticles for the different targets were studied using a Reichert 2 SPR system (Reichert Technologies, Buffalo, USA) with attached autosampler; and Reichert TraceDrawer software,

To immobilize the nanoMIPs, a carboxymethyl dextran hydrogel coated Au chip was preconditioned in PBS for 10 min, then PBST, both at 10 µL min^−1^. Tween was added to limit non‐specific binding. 1 mL of aqueous EDC/NHS solution (40 mg EDC and 10 mg NHS respectively) was then introduced to the chip (6 min at 10 µL min^−1^); followed by 1 mL of 300 µg mL^−1^ of nanoMIPs in PBST (with 10 mm sodium acetate). The materials were incubated in the working channel of the chip for 1 min to immobilize. A quenching solution (1 m ethanolamine, pH 8.5) was then added over both channels for 8 min; followed by a continuous flow of PBST at 10 µL min^−1^ to wash the surface. All injections were taken from a stable baseline.

Here we use an existing rebinding method enabling kinetics of rebinding to be measured.^[^ ^26^ ^]^ Briefly this was a 2‐min association, 5‐min dissociation, and a 1‐min regeneration cycle (RGB) followed by a final stabilization cycle (PBST for 1 min). PBST was used throughout with the association ranges of analyte between 4 and 64 nM; alongside a blank association to baseline zero. In all cases, this measurement was carried out in triplicate.

Signals from the right reference channel were subtracted from signals from the working (left) channel to elucidate the MIP specific binding. The SPR responses were fitted to a 1:1 Langmuir fit bio‐interaction (BI) model. Association rate constants (*k*_a_), dissociation rate constants (*k*_d_), and maximum binding (B_max_) were fitted globally, whereas the BI signal was fitted locally. Equilibrium dissociation constants (K_D_) were calculated from *k*_d_/*k*_a_.

## Conflict of Interest

Aptamer Group has patented the aptamer sequence (Optimer) presented in this work. The academic authors have not conflicts of interest.

## Author Contributions

M.V.S: Investigation, verification, writing—Original draft preparation, methodology; F.A.: Investigation, methodology; H.F.: Investigation, validation; B.B.: Investigation, validation; J.A.R.: Investigation, validation; E.T.B.: Investigation, validation; C.R.: Investigation, validation; P.O.: Investigation, validation; L.J.M.: Investigation, validation; D.B.: Conceptualization, funding acquisition, project administration, writing—Original draft preparation; A.T.: Funding acquisition, project administration; P.M.M.: Funding acquisition, supervision; J.H.R.T.: Supervision, writing—Original draft preparation; N.W.T.: Conceptualization, funding acquisition, project administration, writing—Original draft preparation, methodology, supervision.

## Supporting information

Supporting Information

## Data Availability

The data that support the findings of this study are available from the corresponding author upon reasonable request.

## References

[1] P. Zhou , X. Yang , X. Wang , B. Hu , L. Zhang , W. Zhang , H. Si , Y. Zhu , B. Li , C. Huang , H. Chen , J. Chen , Y. Luo , H. Guo , R. Jiang , M. Liu , Y. Chen , X. Shen , K. Zhao , Q. Chen , F. Deng , L. Liu , B. Yan , F. Zhan , Y. Wang , G. Xiao , Z. Shi , Nature 2020, 579, 270. 32015507 10.1038/s41586-020-2012-7 PMC7095418

[2] A. Raziq , A. Kidakova , R. Boroznjak , J. Reut , A. Opik , V. Syritski , Biosens. Bioelectron. 2021, 178, 113029. 33515985 10.1016/j.bios.2021.113029 PMC7826012

[3] E. J. Snijder , P. J. Bredenbeek , J. C. Dobbe , V. Thiel , J. Ziebuhr , L. L. M. Poon , Y. Guan , M. Rozanov , W. J. M. Spaan , A. E. Gorbalenya , J. Mol. Biol. 2003, 331, 991. 12927536 10.1016/S0022-2836(03)00865-9 PMC7159028

[4] J. Lan , J. Ge , J. Yu , S. Shan , H. Zhou , S. Fan , Q. Zhang , X. Shi , Q. Wang , L. Zhang , X. Wang , Nature 2020, 581, 215. 32225176 10.1038/s41586-020-2180-5

[5] X. Tian , C. Li , A. Huang , S. Xia , S. Lu , Z. Shi , L. Lu , S. Jiang , Z. Yang , Y. Wu , T. Ying , Emerging Microbes Infect. 2020, 9, 382. 10.1080/22221751.2020.1729069 PMC7048180 32065055

[6] D. Wrapp , N. Wang , K. S. Corbett , J. A. Goldsmith , C. L. Hsieh , O. Abiona , B. S. Graham , J. S. McLellan , Science 2020, 367, 1260. 32075877 10.1126/science.abb2507 PMC7164637

[7] W. T. Harvey , A. M. Carabelli , B. Jackson , R. K. Gupta , E. C. Thomson , E. M. Harrison , C. Ludden , R. Reeve , A. Rambaut , S. J. Peacock , D. L. Robertson , Nat. Rev. Microbiol. 2021, 19, 409. 34075212 10.1038/s41579-021-00573-0 PMC8167834

[8] Q. Li , J. Wu , J. Nie , L. Zhang , H. Hao , S. Liu , C. Zhao , Q. Zhang , H. Liu , L. Nie , H. Qin , M. Wang , Q. Lu , X. Li , Q. Sun , J. Liu , L. Zhang , X. Li , W. Huang , Y. Wang , Cell 2020, 182, 1284. 32730807 10.1016/j.cell.2020.07.012 PMC7366990

[9] J. Fantini , N. Yahi , P. Colson , H. Chahinian , B. Scola , D. Raoult , J. Med. Virol. 2022, 94, 2019. 34997962 10.1002/jmv.27577 PMC9015223

[10] Centers for Disease Control and Prevention , https://www.cdc.gov/coronavirus/2019-ncov/variants/variant-classifications.html (accessed: April 2022).

[11] P. Diamandis , I. Prassas , E. P. Diamandis , Clin. Chem. Lab. Med. 2020, 58, 1144. 32386187 10.1515/cclm-2020-0554

[12] N. Kumleben , R. Bhopal , T. Czypionka , L. Gruer , R. Kock , J. Stebbing , F. L. Stigler , Public Health 2020, 185, 88. 32590234 10.1016/j.puhe.2020.06.006 PMC7287442

[13] FDA U.S Food & Drug Administration , https://www.fda.gov/medical-devices/coronavirus-covid-19-and-medical-devices/sars-cov-2-viral-mutations-impact-covid-19-tests (accessed: October 2022).

[14] H. Yu , O. Alkhamis , J. Canoura , Y. Liu , Y. Xiao , Angew. Chem., Int. Ed. Engl. 2021, 60, 16800. 33559947 10.1002/anie.202008663 PMC8292151

[15] N. Zhang , Z. Chen , D. Liu , H. Jiang , Z. K. Zhang , A. Lu , B. T. Zhang , Y. Yu , G. Zhang , Int. J. Mol. Sci. 2021, 22, 4093. 33920991 10.3390/ijms22084093 PMC8071422

[16] C. Alexander , H. S. Andersson , L. I. Andersson , R. J. Ansell , N. Kirsch , I. A. Nicholls , J. O'Mahony , M. J. Whitcombe , J. Mol. Recognit. 2006, 19, 106. 16395662 10.1002/jmr.760

[17] F. Canfarotta , A. Cecchini , S. Piletsky , in Polymer Chemistry Series, (Eds: W. Kutner , P. S. Sharma ), Royal Society of Chemistry, Chichester, UK 2018, pp. 1–27.

[18] F. Canfarotta , A. Poma , A. Guerreiro , S. A. Piletsky , Nat. Protoc. 2016, 11, 443. 26866789 10.1038/nprot.2016.030

[19] H. R. Culver , N. A. Peppas , Chem. Mater. 2017, 29, 5753. 30880872 10.1021/acs.chemmater.7b01936 PMC6420229

[20] G. K. Ali , K. M. Omer , Talanta 2022, 236, 122878. 34635258 10.1016/j.talanta.2021.122878

[21] P. Jolly , V. Tamboli , R. L. Harniman , P. Estrela , C. J. Allender , J. L. Bowen , Biosens. Bioelectron. 2016, 75, 188. 26318788 10.1016/j.bios.2015.08.043

[22] A. O. Rad , A. Azadbakht , Microchim. Acta 2019, 186, 56. 10.1007/s00604-018-3123-9 30617424

[23] M. V. Sullivan , F. Allabush , D. Bunka , A. Tolley , P. M. Mendes , J. H. R. Tucker , N. W. Turner , Polym. Chem. 2021, 12, 4394.

[24] A. Poma , H. Brahmbhatt , H. M. Pendergraff , J. K. Watts , N. W. Turner , Adv. Mater. 2014, 27, 750. 25413444 10.1002/adma.201404235

[25] H. Brahmbhatt , A. Poma , H. M. Pendergraff , J. K. Watts , N. W. Turner , Biomater. Sci. 2016, 4, 281. 26509192 10.1039/c5bm00341e

[26] M. V. Sullivan , O. Clay , M. P. Moazami , J. K. Watts , N. W. Turner , Macromol. Biosci. 2021, 21, 2100002. 10.1002/mabi.202100002 33760365

[27] A. Singhal , A. Parihar , N. Kumar , R. Khan , Mater. Lett. 2022, 306, 130898. 34566219 10.1016/j.matlet.2021.130898 PMC8450140

[28] Z. Bognár , E. Supala , A. Yarman , X. Zhang , F. F. Bier , F. W. Scheller , R. E. Gyurcsányi , Chem. Sci. 2022, 13, 1263. 35222909 10.1039/d1sc04502d PMC8809392

[29] A. G. Ayankojo , R. Boroznjak , J. Reut , A. Öpik , V. Syritski , Sens. Actuators, B 2022, 353, 131160. 10.1016/j.snb.2021.131160 PMC8626155 34866797

[30] F. Puoci , J. Funct. Biomater. 2020, 11, 43. 32560382 10.3390/jfb11020043 PMC7353480

[31] O. I. Parisi , M. Dattilo , F. Patitucci , R. Malivindi , S. Delbue , P. Ferrante , S. Parapini , R. Galeazzi , M. Cavarelli , F. Cilurzo , S. Franzè , I. Perrotta , V. Pezzi , F. Selmin , M. Ruffo , F. Puoci , Nanoscale 2021, 13, 16885. 34528987 10.1039/d1nr03727g

[32] J. McClements , L. Bar , P. Singla , F. Canfarotta , A. Thomson , J. Czulak , R. E. Johnson , R. D. Crapnell , C. E. Banks , B. Payne , S. Seyedin , P. Losada‐Pérez , M. Peeters , ACS Sens. 2022, 7, 1122. 35416035 10.1021/acssensors.2c00100 PMC9016778

[33] MIP Discovery, COVID‐19 nanoMIP, https://mipdiscovery.com/covid19-nanomip (accessed: October 2022).

[34] A. H. M. Safaryan , A. M. Smith , T. S. Bedwell , E. V. Piletska , F. Canfarotta , S. A. Piletsky , Nanoscale Adv. 2019, 1, 379. 10.1039/c9na00327d PMC9419252 36133545

[35] M. V. Sullivan , A. Henderson , R. A. Hand , N. W. Turner , Anal. Bioanal. Chem. 2022, 414, 3687. 35318515 10.1007/s00216-022-04012-8

[36] A. Henderson , M. V. Sullivan , R. A. Hand , N. W. Turner , J. Mater. Chem. B 2022, 10, 6792. 35678703 10.1039/d2tb00270a

[37] N. Caro , T. Bruna , A. Guerreiro , P. Alvarez‐Tejos , V. Garretón , S. Piletsky , J. González‐Casanova , D. Rojas‐Gómez , N. Ehrenfeld , Nanomaterials 2020, 10, 306. 32053989 10.3390/nano10020306 PMC7075134

[38] A. M. Bossi , L. Pasquardini , in Methods in Molecular Biology, Vol. 2359, (Ed: A. Martin‐Esteban ), Springer‐Link, Clifton, NJ, USA 2021, pp. 269–283. 34410676 10.1007/978-1-0716-1629-1_22

[39] S. Jiang , L. Du , Y. He , Y. Zhou , S. Liu , B. J. Zheng , Nat. Rev. Microbiol. 2009, 7, 226. 19198616 10.1038/nrmicro2090 PMC2750777

[40] L. Du , Y. Yang , Y. Zhou , L. Lu , F. Li , S. Jiang , Expert Opin. Ther. Targets 2017, 21, 131. 27936982 10.1080/14728222.2017.1271415 PMC5457961

[41] N. Petrosillo , G. Viceconte , O. Ergonul , G. Ippolito , E. Petersen , Clin. Microbiol. Infect. 2020, 26, 729. 32234451 10.1016/j.cmi.2020.03.026 PMC7176926

[42] D. Frampton , T. Rampling , A. Cross , H. Bailey , J. Heaney , M. Byott , R. Scott , R. Sconza , J. Price , M. Margaritis , M. Bergstrom , M. J. Spyer , P. B. Miralhes , P. Grant , S. Kirk , C. Valerio , Z. Mangera , T. Prabhahar , J. Moreno‐Cuesta , N. Arulkumaran , M. Singer , G. Y. Shin , E. Sanchez , S. M. Paraskevopoulou , D. Pillay , R. A. McKendry , M. Mirfenderesky , C. F. Houlihan , E. Nastouli , Lancet Infect. Dis. 2021, 21, 1246. 33857406 10.1016/S1473-3099(21)00170-5 PMC8041359

[43] H. Tegally , E. Wilkinson , M. Giovanetti , A. Iranzadeh , V. Fonseca , J. Giandhari , D. Doolabh , S. Pillay , E. J. San , N. Msomi , K. Mlisana , A. von Gottberg , S. Walaza , M. Allam , A. Ismail , T. Mohale , A. J. Glass , S. Engelbrecht , G. Van Zyl , W. Preiser , F. Petruccione , A. Sigal , D. Hardie , G. Marais , N. Y. Hsiao , S. Korsman , M. A. Davies , L. Tyers , I. Mudau , D. York , et al, Nature 2021, 592, 438. 33690265 10.1038/s41586-021-03402-9

[44] P. C. Taylor , A. C. Adams , M. M. Hufford , I. de la Torre , K. Winthrop , R. L. Gottlieb , Nat. Rev. Immunol. 2021, 21, 382. 33875867 10.1038/s41577-021-00542-x PMC8054133

[45] S. P. Graham , H. F. El‐Sharif , S. Hussain , R. Fruengal , R. K. McLean , P. C. Hawes , M. V. Sullivan , S. M. Reddy , Front. Bioeng. Biotechnol. 2019, 7, 115. 31179277 10.3389/fbioe.2019.00115 PMC6542949

[46] F. Canfarotta , S. A. Piletsky , N. W. Turner , in Methods in Molecular Biology, Vol. 2073, (Eds: J. A. Gerrard , L. J. Domigan ), Springer‐Link, Clifton, NJ, USA 2020, pp. 183–194. 10.1007/978-1-4939-9869-2_11 31612443

