# Supplementary material for: Highly Selective Aptamer‐Molecularly Imprinted Polymer Hybrids for Recognition of SARS‐CoV‐2 Spike Protein Variants
Source: Glob Chall. 2023 Mar 20;7(6):2200215. doi: 10.1002/gch2.202200215 (PMC10242533; doi:10.1002/gch2.202200215)
Supplement: Supplementary file 1 — Supporting Information [file GCH2-7-2200215-s001.pdf]

Global Challenges
Open Access

Supporting Information

for *Global Challenges,* DOI: 10.1002/gch2.202200215

Highly Selective Aptamer-Molecularly Imprinted Polymer Hybrids for Recognition of SARS-CoV-2 Spike Protein Variants

*Mark V. Sullivan, Francia Allabush, Harriet Flynn, Banushan Balansethupathy, Joseph A. Reed, Edward T. Barnes, Callum Robson, Phoebe O'Hara, Laura J. Milburn, David Bunka, Arron Tolley, Paula M. Mendes, James H. R. Tucker, and Nicholas W. Turner**

Highly Selective Aptamer-Molecularly Imprinted Polymer Hybrids for Recognition of SARS-CoV-2 Spike Protein Variants

*Mark V. Sullivan, Francia Allabush, Harriet Flynn, Banushan Balansethupathy, Joseph A. Reed, Edward T. Barnes, Callum Robson, Phoebe O'Hara, Laura J. Milburn, David Bunka, Arron Tolley, Paula M. Mendes, James H. R. Tucker, Nicholas W. Turner**

M. V. Sullivan, N. W. Turner

Leicester School of Pharmacy, De Montfort University, The Gateway, Leicester, LE1 9BH, United Kingdom

F. Allabush, P. M. Mendes

School of Chemical Engineering, University of Birmingham, Edgbaston, Birmingham, B15 2TT, United Kingdom

F. Allabush, J. H. R. Tucker

School of Chemistry, University of Birmingham, Edgbaston, Birmingham, B15 2TT, United Kingdom

H. Flynn, B. Balansethupathy, J. A. Reed, E. T. Barnes, C. Robson, P. O'Hara, L. J. Milburn, D. Bunka, A. Tolley

The Aptamer Group, Windmill House, Innovation Way, Heslington, York, YO10 5BR, United Kingdom

Email: nicholas.turner@dmu.ac.uk

Supplementary Data (Contents)

Figure S1. BLI sensorgrams showing the concentration dependent binding response of the SARS-CoV-2 S1 proteins to BLI sensor probes consisting of immobilised SARS-CoV-2 Optimer.

Figure S2. BLI sensorgrams show that the aptamer demonstrates preferential binding to SARS-CoV-2 S1 compared to SARS-CoV and MERS-CoV S1 proteins.

Figure S3. Particle size distribution for the SARS-CoV-2 Spike protein S1 Subunit imprinted aptaMIP (A), SARS-CoV-2 Spike protein S1 Subunit imprinted nanoMIPs (B), epitope-imprinted nanoMIPs (C).

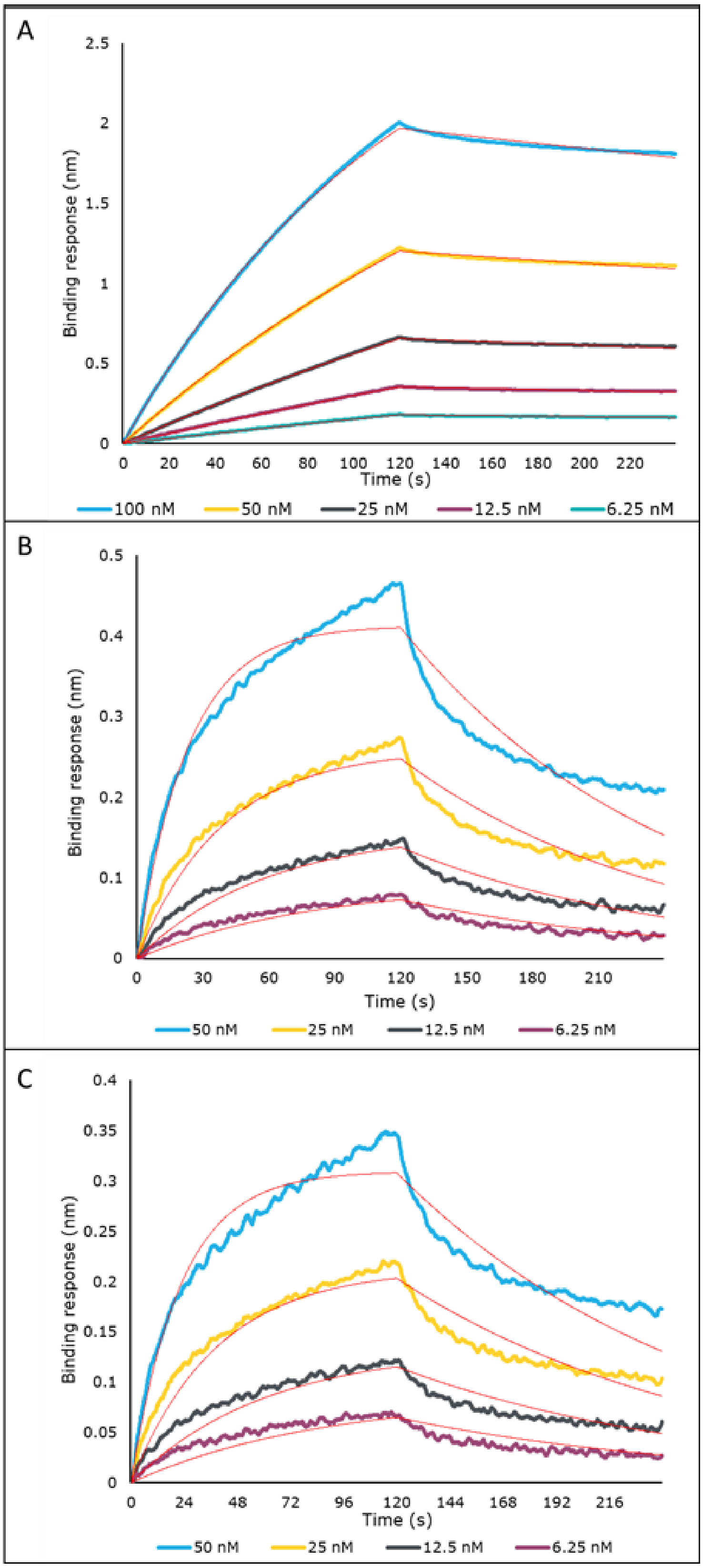

Figure S1: BLI sensorgrams showing the concentration dependent binding response of the SARS-CoV-2 S1 proteins to BLI sensor probes consisting of immobilised SARS-CoV-2 Optimer. Immobilised probes were incubated with (a) SARS-CoV-2 WT, (b) SARS-CoV-2 alpha variant (c) SARS-CoV-2 beta variant. Binding curves were fitted to a global 1:1 binding model (overlays displayed in red).

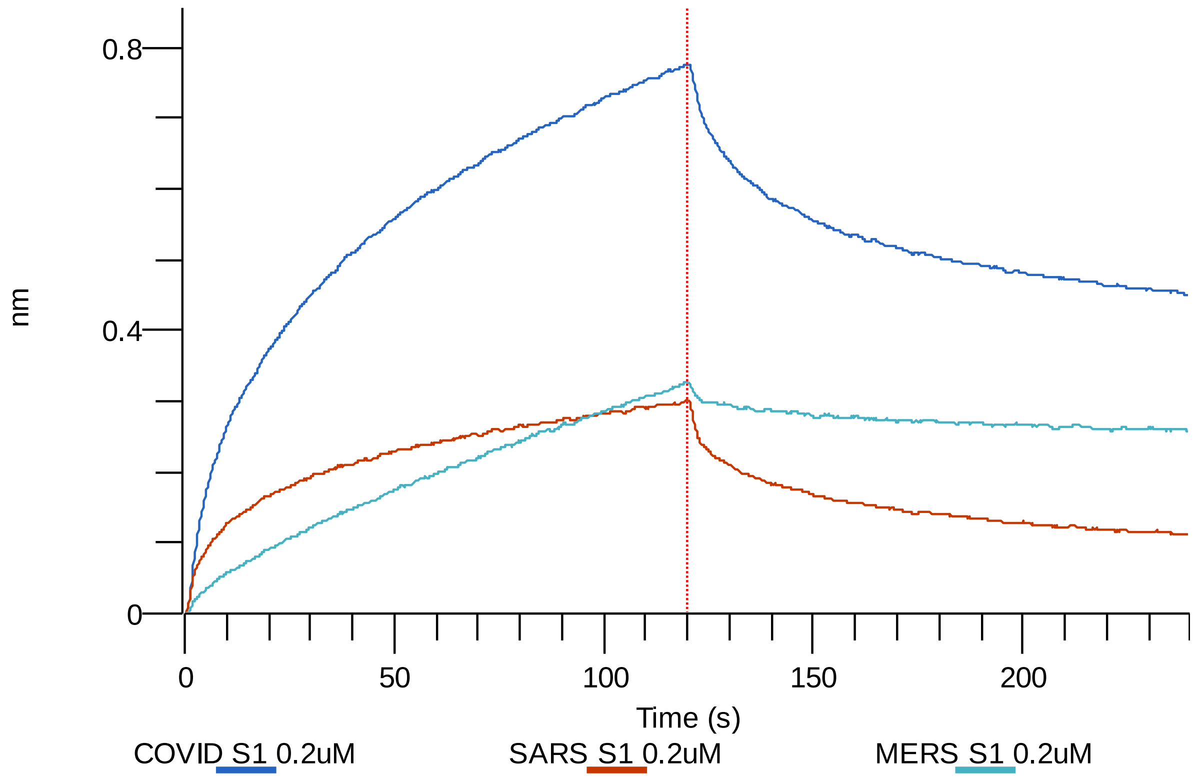

Figure S2: BLI sensorgrams show that the aptamer demonstrates preferential binding to SARS-CoV-2 S1 compared to SARS-CoV and MERS-CoV S1 proteins.

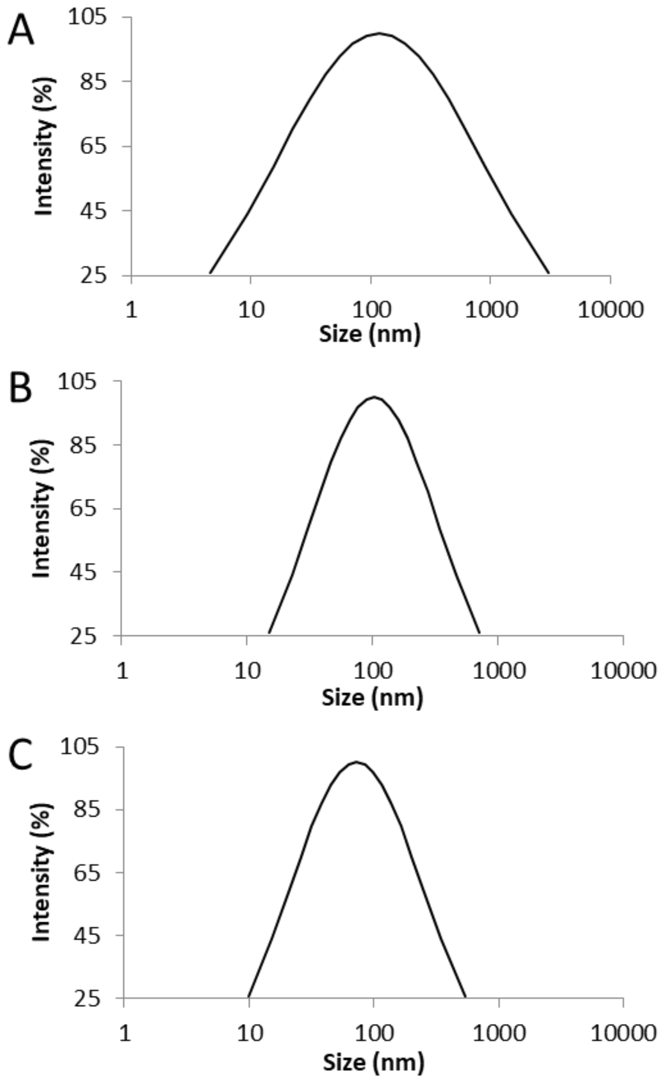

Figure S3: Particle size distribution for the SARS-CoV-2 Spike protein S1 Subunit imprinted aptaMIP (A), SARS-CoV-2 Spike protein S1 Subunit imprinted nanoMIPs (B), epitope-imprinted nanoMIPs (C).

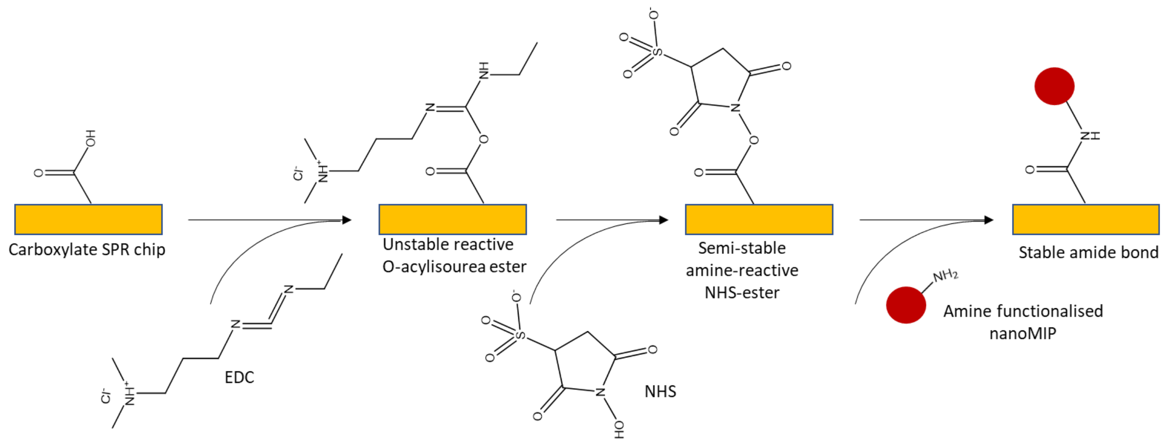

Figure S4. A schematic showing the immobilisation of the amine-functionalised nanoMIP onto a carboxymethyl dextran surface using carbodiimide chemistry.
